# Crocetin Reduces TNBS-Induced Experimental Colitis in Mice by Downregulation of NFkB

**DOI:** 10.4103/1319-3767.54750

**Published:** 2009-07

**Authors:** Hamid A. Kazi, Zhiyu Qian

**Affiliations:** Department of Pharmacology, China Pharmaceutical University, 24 Tongjia Xiang, Nanjing 210009, PR China

**Keywords:** Colitis, crocetin, cytokines, inflammatory bowel disease, NFkB, nitric oxide

## Abstract

**Background/Aim:**

Ulcerative colitis (UC) is characterized by oxidative and nitrosative stress, leukocyte infiltration and upregulation of proinflammatory cytokines. In this study, we aim to investigate the effects of crocetin and its protective mechanism on 2,4,6- trinitrobenzene sulfonic acid (TNBS)-induced colitis in mice model.

**Materials and Methods:**

Intestinal lesions (judged by macroscopic and histological score) were associated with neutrophil infiltration (measured as increase in myeloperoxidase (MPO) activity in the mucosa), and high levels of malondialdehyde MDA (an indicator of lipid peroxidation).

**Results:**

Dose-response studies revealed that treatment of mice with crocetin (50 mg/kg/day) i.g. for 8 days) significantly ameliorated diarrhea and the disruption of colonic architecture. Higher and lower doses (100 and 25 mg/kg/day) did not exhibit comparable effects. In crocetin-treated mice, a significant reduction was noted in the degree of both neutrophil infiltration (measured as decrease in myeloperoxidase activity) and lipid peroxidation (measured as decrease in malondialdehyde activity) in the inflamed colon. Crocetin also reduced the levels of nitric oxide (NO) associated with the favorable expression of TH1 and TH2 cytokines and inducible NO synthase along with the down regulation of nuclear factor-kB (NFkB).

**Conclusion:**

These findings suggest that crocetin exerts beneficial effects in experimental colitis, and therefore we propose that this carotenoid may have therapeutic implications for human UC and can be administered along with the conventional therapy of UC

Inflammatory bowel disease (IBD) consists of Crohn's disease (CD) and ulcerative colitis (UC). Genetic, immunologic and environmental factors are involved in the initiation and perpetuation of chronic intestinal diseases.[1]

Ulcerative colitis (UC) is characterized by oxidative and nitrosative stress, leukocyte infiltration and upregulation of proinflammatory cytokines.[2]

Although the etiology of UC remains unknown, it is believed that exaggerated intestinal immune response plays a key role in the pathophysiology of this intestinal disorder.[3] Treating UC while limiting drug-induced toxicity is a continuous challenge. 5-Aminosalicyclic acid and salazosulfapyridine are the drugs of choice for current medical treatment. Corticosteroids, azathioprine, mercaptopurines and cyclosporine are used in more severe forms of the disease.[4] Crocetin is a constituent of saffron (*Crocus sativus*). Thus far, extensive research has been conducted on crocetin, and it has been shown that it can inhibit tumor promotion,[5] has hepatoprotective activity,[6] is antidiabetic,[7] has antioxidative and anti-inflammatory activities,[8] is useful in cardiac diseases[9] and have antiapoptotic effects.[10] Hence, the objective of this study is to evaluate its effects on colitis and investigate its possible mechanism, as to the best of our knowledge, this has not been done before.

Of the several animal models of intestinal inflammation, the well-characterized haptene reagent TNBS–induced colitis resembles human UC in its various histological features including infiltration of colonic mucosa by neutrophils and macrophages and increased production of inflammatory mediators including TH1 profile of cytokines.[11] Moreover, various experimental trials using antibodies to interleukin (IL)-12 and IL-4 gene transfer, have indicated that the TNBS-induced colitis model is useful to test new therapeutic strategies for humans.[12,13]

## MATERIALS AND METHODS

### Experimental animals and reagents

Female BALB/c mice weighing 25-30 g (Animal Laboratories, Nanjing, China) were housed under normal laboratory conditions of 21°C-24°C and 40%-60% relative humidity, under a 12-h light/dark cycle with free access to standard rodent food and water. All animal procedures were performed in accordance with the institutional guidelines for animal care CPU. The kits for biochemical analysis of NO, MPO and MDA were purchased from Jiancheng Bioengineering Institute (Nanjing, China). Primers for reverse transcriptase-polymerase chain reaction (RT-PCR) were synthesized from Nanjing sunshine company.

### Induction of colitis

Colitis was induced in mice using the modified method described by Morris *et al*., by which 0.1 ml of TNBS/day (60 mg/ml in 30% ethanol), through a trocar needle, using a rubber catheter inserted 3-4 cm via the anus. Mice were kept in vertical position for 30s to prevent solution leakage. Control mice received 0.1 ml of 30% ethanol using the same technique. All the mice were examined daily for body mass and behavior.

### Treatment protocol

To investigate the therapeutic effects of crocetin (≥ 98%, CPU), mice were divided randomly into five groups: control group (n = 10, receiving 30% ethanol only and no treatment) and TNBS group (n = 10 receiving TNBS and no treatment). The treated groups were divided into three groups (n = 10 each) by different dosages of crocetin administered by gavage, i.e., 25, 50 and 100 mg/kg/day to evaluate the effectiveness of different dosages. Crocetin was emulsified by 1% carboxyl methylcellulose before administration. Colitis was induced in treated groups by the same technique mentioned above. The model was established for 8 days. Spleen and colon weights of all mice were calculated after killing them.

### Assessment of severity of colitis

#### Assessment of biochemical parameters

After rapid removal of the colon, specimens were flushed with ice-cold saline, cut open, photographed and were scored by an observer unaware of the treatment, by using following scoring system of Wallace and keenan:[14] 0) no damage, 1) localized hyperemia without ulcers, 2) linear ulcers with significant inflammation, 3) linear ulcer with inflammation at one site, 4) two or more sites of ulceration and/or inflammation, 5) two or more major sites of inflammation and ulceration extending more than 1 cm along the colon. For histological analysis, tissues were fixed in 100 g/L paraformaldehyde in phosphate-buffered saline and paraffin-embedded tissue sections were stained with hematoxylin and eosin (H&E) using the standard techniques

#### Assessment of NO production

Increased luminal activities of NO have been detected in UC.[15] Therefore, Interventions, which reduce the generation of reactive nitrogen, exert beneficial effects in colitis.[16,17] Tissues from the proximal third of the colon were homogenized in 40 mM HEPES containing 320 mM sucrose. Nitrite + nitrate production, an indicator of NO synthesis, was measured in the supernatant (10,000 *g* for 20 min at 4°C) according to the method described by Zingarelli *et al*. (1997). Nitrate in the supernatant was reduced to nitrite by incubation with nitrate reductase (670 mU/ml) and NADPH (160 mM) at room temperature for 3 h. A measure of 100 ml of the sample was then mixed with an equal volume of Griess reagent (1% sulfanilamide and 0.1% N-(1-napthyl)-ethylenediamine dihydrochloride in 5% H_3_PO_4_) and incubated at room temperature for 10 min. Absorbance was then measured at 540 nm. The amount of nitrite released was quantified by comparison with sodium nitrite as standard

#### Assessment of leukocyte infiltration

Myeloperoxidase is an enzyme found in cells of myeloid origin, and has been used extensively as a biochemical marker of granulocyte (mainly neutrophil) infiltration into gastrointestinal tissues.[18] Samples of distal colon were homogenized in 10 mM potassium phosphate buffer, pH 7.0 containing 0.5% hexadecyltrimethylammonium bromide and centrifuged for 30 min at 20,000*g* at 4°C. An aliquot of the supernatant was then allowed to react with a solution of 1.6 mM tetramethyl benzidine and 0.1 mM H_2_O_2_. The rate of change in absorbance was measured spectrophotometrically at 650 nm. One unit of myeloperoxidase activity was defined as the amount degrading 1 mmol of H_2_O_2_ per minute at 37°C and was expressed as units per milligram of tissue sampled (U/mg tissue).

#### Assessment of lipid peroxidation

Malondialdehyde levels in the colon were determined as an indicator of lipid peroxidation.[19] The tissue was homogenized in 1.15% KCl solution. A measure of 0.1 ml of the homogenate was then added to a reaction mixture containing 0.2 ml of 8.1% sodium dodecyl sulfate, 1.5 ml of 20% acetic acid, 1.5 ml of 0.8% thiobarbituric acid and 0.7 ml of distilled water. Samples were boiled for 1 h at 95°C and centrifuged at 3000*g* for 10 min. The absorbance of the supernatant was measured by spectrophotometry at 650 nm.

RT-PCR analysis of cytokines and inducible nitric oxide synthase (iNOS) mRNA Mice colons were snap-frozen in liquid nitrogen and stored at −70°C. Total RNA was isolated by the TRIzol (Invitrogen). A 260/280 ratio was evaluated to examine the quality of RNA. Subsequently, total RNA was reverse transcribed into complementary DNA (cDNA) using a first strand cDNA kit from (Nanjing sunshine company, China). Later, PCR was carried out in an automatic DNA thermal cycler (BioRad technologies). For amplification of the desired cDNA, gene-specific primers [Table 1] were used. The PCR products were electrophoresed on 2% agarose gels, stained with 0.5 mg of ethidium bromide, and observed with a UV transilluminator. Sequences of the oligonucleotide primers used for PCR amplification of cytokine and iNOS are as follows.

**Table 1:** Colonic erosion scale

	0	1	2	3	4	n	Mean	*P*
Control	6	4	0	0	0	10	0.40	-
TNBS	0	1	3	3	3	10	2.80	-
crocetin (25 mg/kg)	0	2	4	2	2	10	2.40	0.4372
crocetin (50 mg/kg)	0	7	2	1	0	10	1.40	0.0052
crocetin (100 mg/kg)	0	7	3	0	0	10	1.30	0.0021

**IL-4**

Sense: 5'-TAGTTGTCATCCTGCTCTT-3' 404 bp

Antisense: 5'-CTACGAGTAATCCATTTGC-3'

**IL-12p40**

Sense: 5'-CAGAAGCTAACCATCTCCTGGTTTG-3' 394bp

Antisense: 5'-TCCGGAGTAATTTGGTGCTTCACAC-3'

**Interferon-**γ

Sense: 5'-AACGCTACACACTGCATCT-3' 342 bp

Antisense: 5'-TGCTCATTGTAATGCTTGG-3'

**iNOS**

Sense: 5'-CATGGCTTGCCCCTGGAAGTTTCTCTT CAAAG-3' 754 bp

Antisense: 5'-GCAGCATCCCCTCTGATGGTGC CATCG-3'

### Electrophoretic mobility shift assay

The nuclear extracts were prepared from excised colon according to the method of Yang *et al*.[20] For electrophoretic mobility shift assay, every 10 mg of nuclear extracts were preincubated with 1 mg of poly(dI-dC) in a binding buffer (25 mM HEPES, pH 7.9, 0.5 mM EDTA, 0.5 mM dithiothreitol, 1% Nonidet P-40, 5% glycerol and 50 mM NaCl) for 10 min at room temperature. As a control, a 50-fold molar excess of unlabelled NFkB competitor oligonucleotide was added. After preincubation, 0.5 ng of 32P end-labelled NFkB oligonucleotide probe (5'–GGACTTTCCGCTGGGGACTTTCCGCTT GAGCT-3') was added to the reaction mixture and incubated for 30 min. The DNA-protein complex was then electrophoresed on 4.5% nondenaturing polyacrylamide gels in 0.5X TBE buffer (0.0445 M Tris, 0.0445 M borate, 0.001 M EDTA).

### Statistical analysis

Results are expressed as mean ± S.D. of n observations. We used analysis of variance to determine the statistical significance of intergroup comparisons. *P* < 0.05 was considered be statistically significant.

## RESULTS

### Body, colon and spleen weight

**Figure 1:**
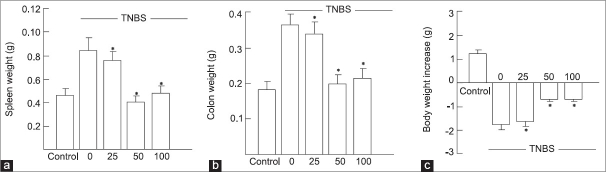
Effect of crocetin treatment on (a) spleen, (b) colon and (c) body weight. Values are means±s.d. of 10 mice for each group. *P< 0.01 vs TNBS.

A significant increase in spleen and colon weight and decrease in body weight was observed after TNBS administration. Crocetin treatment (25, 50 and 100 mg/kg/day) significantly prevented the loss in body weight (c) as well as reduced the organ weight (a and b) (data presented by graphs only). Crocetin 50 mg/kg seems to be the most effective treatment [Figure 1].

### Macroscopic score and histological images

**Figure 2:**
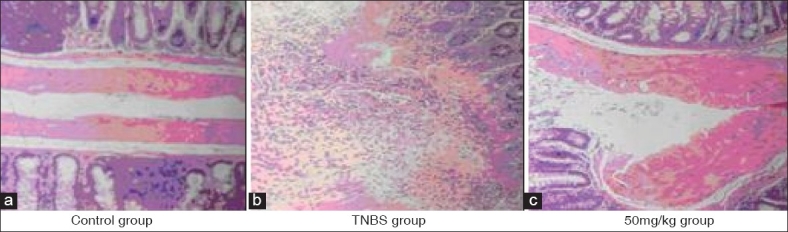
HE staining for histological assessment (a) no sign of damage in colon (b) severe mucosal damage, leukocyte infiltration visible (c) edema of colon, but no mucosal damage or leukocyte infiltration

TNBS-induced colitis was characterized by thick and stiff colonic wall as a result of edema, chemical erosion or fibriform-proliferation. Ulcers were scattered along the colon or linked with each other, bleeding or redness was observed in whole or partial colon. Necrosis of epithelium, distortion of crypts, destruction of glands and infiltration of inflammatory cells were observed. Severity of colitis in crocetin 50 mg/kg was significantly lower than that of TNBS. Treatment with crocetin decreased edema. Ulcers in crocetin-treated groups were much smaller and superficial; most of the ulcers were healed and granulation or fibroplasias could be seen. Histological analysis showed that crocetin treatment decreased necrosis of epithelium and the infiltration of inflammatory cells [Table 1 and Figure 2].

### Generation of NO

**Figure 3:**
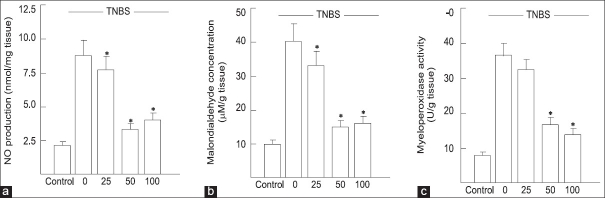
(a) Effect of crocetin on NO production in colonic tissue Values are means ± s.d. of 10 mice for each group. *P< 0.01 vs TNBS. (b,c) Effect of crocetin on myeloperoxidase activity and malondialdehyde levels in the colon. Values are means ± s.d. of 10 mice for each group. *P< 0.01 vs TNBS

After induction of colitis, the nitric oxide level in the TNBS group (8.8 ± 1.0 nmol/mg tissue) were significantly elevated compared to control group (2.0 ± 0.3 nmol/mg tissue) (crocetin treatment at dose levels of 25, 50 and 100 mg/kg/day resulted in marked decrease in the elevated levels of NO [Figure 3a] in the colon of TNBS-treated mice (*P* < 0.01). A dose of 50 mg/kg was most effective.

### Effect of crocetin on myeloperoxidase and malondialdehyde levels in TNBS-induced IBD

Colonic injury by TNBS administration was also characterized by an increase in myeloperoxidase activity (36.7 ± 3.3 in TNBS group compared with 8.0 ± 1.3 U/g tissue in control group), indicative of neutrophil infiltration in inflamed tissue [Figure 3b], confirming the enhanced leukocyte infiltration seen at histological inspection. In this study, the extent of myeloperoxidase activity closely paralleled the increase of tissue malondialdehyde, (40.7 ± 4.1 in TNBS group compared with 9.3 *±* 1.4 *μ*M/g tissue in controls), indicative of a massive lipid peroxidation [Figure 3c]. However, crocetin treatment of TNBS-treated mice at dose levels of 25, 50 and 100 mg/kg/day significantly prevented neutrophil infiltration, as assessed by myeloperoxidase activity (*P* < 0.01) and also prevented the increased accumulation of malondialdehyde (*P* < 0.01) and 50 mg/kg dose yielded the best efficacy because of unknown reason.

### Cytokine production in treated mice

**Figure 4:**
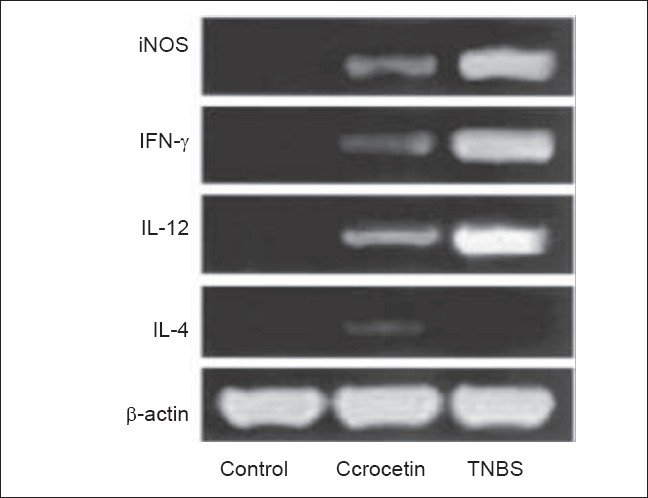
Suppression of Th1 phenotype in TNBS-treated mice subjected to crocetin treatment (50 mg/kg) as analysed by RT – PCR. (a) Expression of IFN-γ, IL-12, IL-4, iNOS and β-actin mRNA by colon tissue samples of control and treated mice. β-actin expression levels were used as controls for RNA content and integrity

We examined the mRNA expression for a representative TH1 cytokine (e.g. interferon [IFN]-γ), a TH1 inducer (e.g. IL-12), a TH2 cytokine (IL-4) and iNOS, which catalyses the generation of NO from L-arginine and plays a major role in colitis. RT-PCR analysis of cytokine mRNA levels confirmed that experimental colitic mice treated with crocetin could reverse an established TH1 response into a possible TH2 response [Figure 4]. Thus, mucosal cells from mice treated with TNBS contained significantly increased levels of IFN-γ and IL-12 p40 mRNAs than those from control group representing a dominant inflammatory TH1 response. However, crocetin treatment resulted in marked suppression of both IFN-γ and IL-12 p40 mRNA levels with a slight induction of IL-4 mRNA in TNBS-treated mice. In addition, the iNOS mRNA expression, which was very high in the mucosal cells of TNBS-treated mice, was significantly decreased by crocetin treatment. These results suggest that inflammatory TH1 functions have been effectively suppressed in BALB/c mice by crocetin treatment so that TH2 functions could possibly be activated to ameliorate mucosal injury in experimental colitis.

### NFkB in colonic mucosa of treated mice

**Figure 5:**
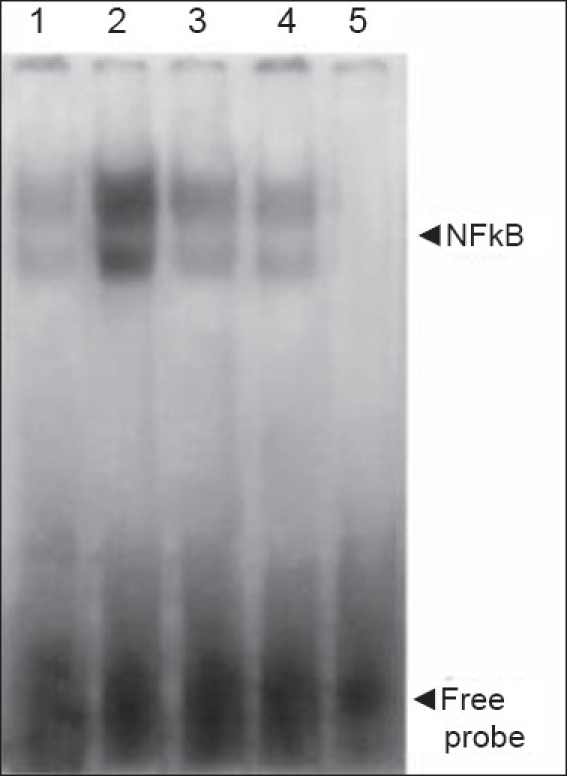
Effect of crocetin treatment on NF-kB activation. Nuclear extracts of the colonic tissue from control mice (lane 1), untreated mice with TNBS-induced colitis in absence (lane 2) and presence of 50 molar excess of unlabelled probe (lane 5) and crocetin (100 and 50 mg/kg) treated mice with TNBS-induced colitis (lane 3 and 4) were analysed

The administration of TNBS alone enhanced NFkB DNA-binding activity of nuclear extracts in the inflamed colonic tissue, which was suppressed by treatment with 50 mg/kg/day crocetin [Figure 5]. Excess unlabelled specific oligonucleotides inhibited NFkB mobility shift, indicating the specificity of DNA-protein complex.

Nuclear extracts of the colonic tissue from control mice (lane 1), untreated mice with TNBS-induced colitis in the absence (lane 2) and presence of 50 M excess of unlabelled probe (lane 5) and crocetin (100 and 50 mg/kg)-treated mice with TNBS-induced colitis (lanes 3 and 4) were analyzed.

## DISCUSSION

The use of natural anti-inflammatory products provides an attractive and relatively nontoxic alternative to modulate inflammatory disorders.[21] Crocetin C_20_H_24_O_4_, a major ingredient originally found in the dried stigma of Crocus sativus L (saffron). has been used in the treatment of diversiform diseases for a long time[22] and it has anti-inflammatory effects.[23] The present study has demonstrated that TNBS causes a substantial degree of inflammation and tissue injury in the mouse colon, by infiltration of the colon with polymorphonuclear cells (histology and myeloperoxidase activity) as well as lipid peroxidation. The degree of inflammation, tissue injury and lipid peroxidation caused by TNBS was substantially reduced in mice treated with all the doses of crocetin but of all the doses 50 mg/kg/day crocetin dose was most effective. After treatment, the cytokine profile in these mice indicated a switch from proinflammatory TH1 to anti-inflammatory TH2 pattern. Reactive NO radical is known to play a central role in human IBD. Increased production of NO, and the presence of iNOS protein and iNOS mRNA have been demonstrated in affected areas of the gut in patients suffering from UC or Crohn's disease.[24–26] Prolonged production of high amounts of NO by iNOS is proinflammatory and inhibition of iNOS seems to ameliorate the inflammatory response and tissue injury in experimental colitis model. An *in vivo* study by McKenzie *et al*.[27] provides direct evidence on NO-induced injury on gut epithelial cells, supporting the detrimental role of excessive NO in colitis. There is, therefore, good rationale to suggest that inhibition of excessive NO production by iNOS inhibitors will serve as promising approach in the management of IBD. We provide here the in vivo evidence that NO concentrations can be downregulated via suppression of proinflammatory cytokines by crocetin in TNBS-induced colitis, resulting in a significant amelioration of the disease. There is ample evidence in human IBD that the inflammatory cytokines such as IL-1 and IFN-γ are overexpressed and this finding correlates with reports of excessive amounts of NO produced by activated iNOS in lamina propria mononuclear cells and colon epithelial cells.[25,28] This prompted us to investigate whether manipulation of cytokine profile by crocetin would lead to reduced NO activities, and thus decrease mucosal damage. Crocetin treatment led to a marked suppression in IL-12 mRNA expression by mucosal cells of TNBS-administered mice, resulting in a reduced ability to induce IFN-γ and perhaps, an increased ability to induce IL-4 in CD4+ T cells.

These results suggest that crocetin-mediated inhibition of IL-12 production led to the inhibition of TH1 and a possible enhancement of TH2 cytokine synthesis in CD4+ T cells. The mechanism by which crocetin inhibits IL-12 production is through the downregulation of NFkB-mediated activation and binding to the p40-kB site as crocetin can inhibit NFkB activity in electrophoretic mobility shift assay, using nuclear extracts of whole cells of the colonic tissue. NFkB activation is believed to play a major role in the regulation of proinflammatory gene transcription; therefore, suppression of NFkB activation crocetin may inhibit early steps of inflammation and modulate upregulation of multiple proinflammatory genes.

The curative effect of crocetin accompanied by reduced levels of nitric oxide and malondialdehyde is suggestive of the scavenging capability of both reactive nitrogen and oxygen species by this natural carotenoid. These results are consistent with previous data, indicating that certain carotenoids are capable of lowering the generation of both superoxide and nitric oxide from rat peritoneal macrophages.[29] All these observations may have relevance on the beneficial effect of oral administration of crocetin in inflammatory disease of the bowel. In conclusion, this study demonstrates that the degree of colitis caused by administration of TNBS is significantly attenuated by crocetin. The anti-inflammatory effects of crocetin are associated with a reduction in (i) upregulation of proinflammatory TH1 cytokine response leading to the suppression of iNOS and attenuation of the recruitment of neutrophils, (ii) lipid peroxidation and (iii) ultimately, tissue injury. Being a relatively nontoxic natural product, combined with its excellent anti-inflammatory activity via reducing inflammatory cytokines, iNOS and NFκB downregulation, crocetin could be useful in human IBD as a supplement therapy.
